# Application of mendelian randomization in ocular diseases: a review

**DOI:** 10.1186/s40246-024-00637-1

**Published:** 2024-06-17

**Authors:** Xiran Zhang, Weichen Yuan, Jun Xu, Fangkun Zhao

**Affiliations:** 1 China Medical University, Shenyang, China; 2 Department of Ophthalmology, The Fourth Affiliated Hospital of China Medical University, Shenyang, China; 3 Key Lens Research Laboratory of Liaoning Province, Shenyang, China; 4 Department of Ophthalmology, Shenyang the Fourth People’s Hospital, Shenyang, China

**Keywords:** Mendelian randomization, Ocular disease, Risk factor, Etiological research, Casual relationship

## Abstract

Ocular disorders can significantly lower patients’ quality of life and impose an economic burden on families and society. However, for the majority of these diseases, their prevalence and mechanisms are yet unknown, making prevention, management, and therapy challenging. Although connections between exposure factors and diseases can be drawn through observational research, it is challenging to rule out the interference of confounding variables and reverse causation. Mendelian Randomization (MR), a method of research that combines genetics and epidemiology, has its advantage to solve this problem and thus has been extensively utilized in the etiological study of ophthalmic diseases. This paper reviews the implementation of MR in the research of ocular diseases and provides approaches for the investigation of related mechanisms as well as the intervention strategies.

## Introduction

Identification of causal factors are crucial for treatments of ocular diseases, which benefits both individual and societal perspectives. Randomized controlled trials (RCTs) have been likened to the “gold standard" [1]for establishing causality and have been shown to have an elevated level of evidence, but they are constrained by their costly nature and potential ethical problems. In observational studies, it is simple to infer relationships between environmental factors and disease, whereas it often proves difficult to demonstrate causation. This is due to three key factors: (1) There are potential interfering factors in the external environment that have not been controlled; (2) Confounders lead to “pseudo-cause and effect” cannot be avoided; (3) Reverse causation cannot be ruled out since causal association encompasses both going from cause to effect and from effect to cause. Despite confounders can be removed via regression modeling, it might be hard to obtain totally unbiased findings from these analyses if they are not noticed or a mast. At the same time, the dependent variable changes simultaneously with the independent variable due to reverse causation, which prevents traditional regression analyses from yielding findings.

To avert impractical outcomes caused by RCTs or observational researches, Mendelian randomization (MR), an epidemiological and genetics-based research method, has been welcomed in etiological researches.

### Core of MR

**Fig. 1:**
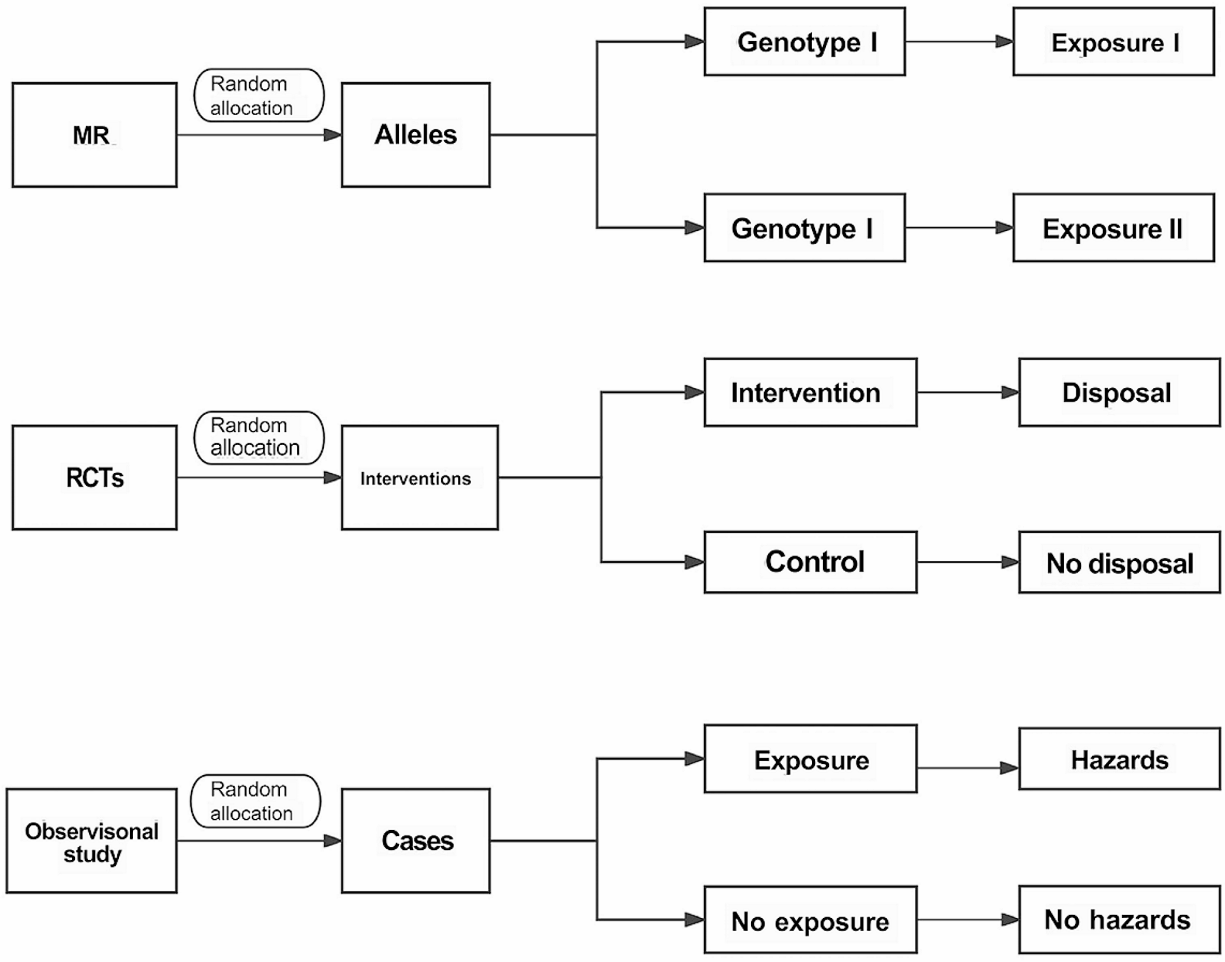
The relationship and distinctions among the three studies. Mendelian randomization (MR) (1): This technique substitutes genetic allels assigned randomly for population-randomized allocation (2). Randomized controlled trial (RCT): RCTs use randomization to split the population into different subgroups with diverse interventions (3). Observational study: It has several types whose core is a division of groups based on whether or not they are exposed to a certain research factor in order to examine the risk factor

Genes highly associated with specific traits as instrumental variables (IVs) [2] are highly used in MR to replace exposures in the regression model to indirectly determine the causal relationship between exposure and outcome. This method is used to assess the causal relationship between exposures and clinical outcomes in observational studies. In contrast to observational studies, MR avoids confounding, reverse causality, selection bias, and error attenuation. Since genes are randomly assigned at the time of gamete formation, they are hardly affected by the external environment after birth, and have only a unidirectional relationship with exposure [3]. The relationship and distinctions among MR, RCT and observational studies were shown in Fig. 1.

### How bias is avoided by MR

The IVs must meet the following three requirements for MR to achieve successful unbiased estimation: (1) it is necessary for the instrumental variables to be correlated with the exposure; (2) they cannot be linked to confounders; (3) they cannot directly affect the outcome variables and can only be linked to them through the exposure. The first two conditions can be achieved by genome-wide association studies (GWAS) using large sample size; however, the third condition is more challenging to obtain and is more likely to have greater errors due to the potential spread of confounding factors through gene pleiotropy or chain disequilibrium. In actual practice, sensitivity analysis, inverse variance weighting (IVW), plurality scoring [4] etc. are typically used to account for bias [5].

### Clinical MR design

One-sample MR and two-sample MR are currently the most popular clinical design concepts. The key distinction between them is the sample size of the patient population: the latter is employed more frequently due to its greater sample size and ease of use of GWAS data. In addition, bi-directional MR analyses are used to ascertain the direction of causation between exposures and outcomes; network MR, represented by two-step MR, is employed to find potential pathogenic mechanisms in the causation between exposures and outcomes; multivariate MR analyses are employed to find associations between numerous exposures with related effects and to investigate the causative values of numerous exposure factors, etc. The visualization of these MR analyses can be further achieved by creating funnel plots, Q-contribution plots, scatter plots, radial plots, and forest plots.

## MR in ocular diseases

### Conjunctivitis

Allergic conjunctivitis (AC), a common ocular disease caused by allergens, may lead to symptoms like itching, tears, increased secretions, conjunctival congestion, photophobia, etc. and can subside itself by detaching from the allergen. Some people have allergies, which can lead to a number of allergic conditions including eczema and allergic rhinitis. In previous studies AC is considered linked with common allergens like climate, diet as well as pollen dispersion [6], microbial exposures like tuberculosis [7]. Recent MR is utilized to investigate how certain diseases, microorganisms and metabonomics affect allergic conjunctivitis. Zhou et al. [8]designed a bidirectional two-sample MR analysis (allergic conjunctivitis: OR, 1.53; atopic conjunctivitis: OR, 1.76; chronic conjunctivitis: OR, 1.76), and found that atopic dermatitis is a cause of conjunctivitis while conjunctivitis does not cause atopic dermatitis, establishing the causal link between the two. By using techniques like inverse variance weighted (IVW) estimation, Zhang et al. ultimately reached their conclusion that attention deficit hyperactivity disorder was not related to allergic conjunctivitis [9]. Since previous observational studies show growing evidence that gut microbiota associate with the incidence of AC closely, Liu et al. came to the conclusion that gut microbiota may be involved in immunomodulation and lower the incidence of allergic conjunctivitis, except for Oscillospira flora, which produces substances linked to inflammatory diseases and contributes to the incidence of allergic conjunctivitis [10]. To further estimate genetic associations between polyunsaturated fatty acids and allergy disorders, a two-sample MR analysis was used to identify that: omega-3 fatty acids had a preventive effect on the development of AC, while omega-6:3 fatty acids raised the risk of AC, taking into account age, economic status, BMI, and daily behavioral habits [11]. The identification of these correlates offers a novel perspective on the etiology of allergic conjunctivitis as well as suggestions for targeted prevention and treatment strategies [9].

### Age-related cataract

Cataract is the leading cause of blindness in the world, accounting for around 51% of all ocular cases [12]. The symptoms consist of decreasing visual acuity, impaired contrast sensitivity, and eventually lead to total blindness as the condition worsens.

Lifestyle has long been acknowledged as a significant contributor to age-related cataracts. An 11-year follow-up observational study indicated that poor lifestyle habits including smoking and drinking alcohol resulted in an increased morbidity of cataracts at a younger age, which led to the conclusion that these bad lifestyle choices contributed to the formation of cataracts [13]. In an MR study, Yuan et al. also concluded that type 2 diabetes mellitus, high systolic blood pressure, and smoking increased the risk of cataracts in the elderly [14]. However, a two-sample MR conducted by Chen et al. showed that BMI, type 2 diabetes mellitus and hypertension were not linked to the development of cataracts, and there was insufficient evidence that smoking or alcohol intake was linked to the ailment [15]. The aforementioned biases might be attributed to various testing techniques, statistical methods, sample sizes, which implies that a more logical experimental design is required to mitigate these errors.

According to different positions in lens changes, age-related cataracts can be divided into three types, namely cortical cataracts, nuclear cataracts and posterior cataracts. An observational study conducted by Johns Hopkins University revealed that diabetes and high systolic blood pressure were risk factors for posterior subcapsular cataracts (RR 2.2 and RR 6.6, respectively) [16]. Lim et al. found that obesity was associated with both posterior and cortical subcapsular cataracts by using MR analysis [17]. But neither of them identified relationships between lifestyle and nuclear cataracts.

### Glaucoma

Glaucoma is a collection of illnesses with the character of elevated intraocular pressure, leading to progressive optic nerve damage and visual field abnormalities. It is the main contributor to irreversible blindness and the second most frequent cause of blindness after cataracts [18]. Primary glaucoma, which includes the common open-angle and closed-angle glaucoma, is distinguished from secondary glaucoma and congenital glaucoma. The goal of treatment is to lower intraocular pressure (IOP) [19], which is primarily split into two categories: commonly used eye drops like β-blockers, carbonic anhydrase inhibitors, pilocarpine, prostaglandin derivatives, etc., which are capable of lowering IOP by increasing atrial aqueous humor efflux and decreasing atrial aqueous humor production; Surgery consists of laser procedures such trabeculoplasty, peripheral iridectomy [20], and filtration surgery with extraocular drainage. Ginkgo biloba extract, which protects the optic nerve [21], and hyperbaric oxygen therapy, which lowers IOP [22], are further treatments.

#### Lipid synthesis

There are many controversies in the observational study of glaucoma, and the impact of aberrant lipid metabolism on glaucoma is an explosive research field [23–25]. By using MR strategies, Xu et al. concluded that there is no causal connection between LDL, HDL, TG [26]and primary open-angle glaucoma (POAG). According to Nusinovici et al., normally tested lipids (HDL, LDL, etc.) were not linked with POAG, but HDL3 was strongly associated with POAG. To be specific, the greater level of HDL3, the lower incidence of POAG is [27]. Bao et al. found that DHA and AA (arachidonic acid) etc. were not related to POAG after conducting a two-sample MR analysis on the gene sequences of 216,257 European participants [28]. The impact of aberrant lipid metabolism on the disease should be continuously monitored, and further investigation into the connection between plasma lipoproteins and glaucoma is still essential.

#### Coffee consumption

Given the high percentage of caffeine in tea, coffee, and other beverages, it is crucial to understand how caffeine affects IOP in patients with glaucoma. It has been demonstrated in a placebo-controlled, double-blind research that individuals who do not habitually use caffeine may have an abrupt spike in IOP after consuming caffeine [29]. One cup of coffee (182 mg of caffeine) had no appreciable impact on IOP in healthy subjects or participants with a family history of glaucoma, according to a randomized controlled experiment [30]. According to Li et al.’s findings from a two-sample MR study, the intake of caffeine is genetically linked to POAG, and the more caffeine is taken in, the more likely one is to suffer from POAG [31]. Data from the UK Biobank were subjected to an MR analysis by Kim et al., who came to their view that while habitual caffeine consumption does not raise the risk of glaucoma, it does raise IOP in individuals with susceptibility genes and contributes to the risk of open-angle glaucoma in those with a family history of genetic diseases [32]. In conclusion, people with a family history of glaucoma should check their IOP and pay attention to how much caffeine they consume.

#### Myopia

Myopia has been implicated in numerous studies as an important risk indicator for glaucoma. A meta-analysis revealed that myopic individuals have a likelihood of developing POAG which is roughly double that of non-myopic patients [33]. Another investigation revealed a link of OR 1.88 between myopia and POAG, elevating the incidence of glaucoma by 20% for every increase in myopia of 1.00 D [34]. By using a two-sample MR analysis, Choquet et al. concluded that myopic patients have a greater likelihood of developing POAG and that there is a causal relationship between the two. Meanwhile, in comparison with the control group, patients with POAG present a higher proportion of high myopia because these two diseases share common genes [35]. Chong et al. uncovered genetic relations between myopia and POAG, and suggest that reduce IOP, the mediator, may help myopia control [36].Therefore, glaucoma can be prevented or postponed to some extent by controlling and preventing myopia.

### Uveal diseases

Uvea, the intermediate layer of the eye, includes iris, ciliary body, and choroid. The uvea is vulnerable to be attacked by the autoimmune system and manifests as uveitis due to its high and slow blood flow, richness in melanin-associated antigens.

#### Anterior uveitis

The most typical type of uveitis is anterior uveitis, encompassing iritis, iridocyclitis, and anterior cyclitis. Genetic factors are significant contributors to the etiology of anterior uveitis. There is proof that the human leukocyte antigen HLA-B27 and acute anterior uveitis are closely related [37]. Meng et al. came to the conclusion through a bidirectional MR design that both Crohn’s disease and ulcerative colitis were associated with iridocyclitis. And Crohn’s disease tends to be stronger related to iridocyclitis than ulcerative colitis, which suggests that patients with inflammatory bowel disease undergo regular eye examinations for early detection is necessary [38]. By summary level MR data, Shu et al. came to the conclusion that linoleic acid inhibits juvenile idiopathic arthritis associated iridocyclitis, but arachidonic acid leads to its development [39]. Using a two-sample MR design and four bias correction techniques, Lin et al. eventually reached the conclusion that low TIM-3 expression levels might be protective against anterior uveitis [40]. However, further research and computations are still required. These discoveries open up new avenues for the etiologic treatment of anterior uveitis and add to the possibilities for its pathogenesis.

#### Behcet’s disease

Behcet’s disease is marked by recurrent uveitis, oral and vaginal ulcers, and skin lesions and may lead to multisystem autoimmune conditions. Although it has unclear pathophysiology, certain research has indicated that its most significant susceptibility gene is HLA-B5/B*51 [41]and those with genetic susceptibility are more sensitive to infections and environmental triggers [42]. By combining IVW and the tuberculin test, Zhong et al. obtained that tuberculosis infection was a separate risk indicator for Behcet’s disease in patients with a history of uveal illness, with an OR of 2.26 [43]. By using IVW, Zhong et al. conducted two cohort studies in China and Turkey and came to the conclusion that long-term high 25(OH)D levels would increase the risk of Behcet’s disease (OR 3.96) [44]. Currently, at the therapeutic level, the use of interleukins and receptors like IFN-α and TNF-α can significantly enhance the prognosis of patients [45]. It is believed that with the further elucidation of its pathogenesis, there will be great development in the treatment of the cause.

### Retinopathy

#### Age-related Macular Degeneration (AMD)

One of the most frequent causes of irreversible, long-term impairment of vision is age-related macular degeneration (AMD). Most of the patients are over 50 years old, and they typically complain about visual impairment, blurred vision, and black shadows. It can be classified into dry and wet forms based on clinical symptoms and macular pathological alterations, and both eyes may develop concurrently or successively. Neovascular age-related macular degeneration (NVAMD), characterized by an abrupt loss of vision in a short period of time (weeks or months), is a more serious type preceded by the development of aberrant blood vessels in the macular. Vascular endothelial growth factor A inhibitors, such as pegaptanib, bevacizumab, ranibizumab, and abciximab [46], are injected as the main kind of treatment. Additionally, multi-targeted treatments like OPT-302 and the bispecific Ang2-VEGFA antibody are being researched [47].

In the early and middle stages of the disease, dry age-related macular degeneration may only manifest a limited number of vitreous membrane warts and no abnormal visual sensation; in the late stage, it may progress to macular geographic atrophy, and the patient’s visual acuity of both eyes gradually declines. Antioxidant therapy, neuroprotective drugs, gene therapy, and C5 inhibitors all work to reduce geographic atrophy [48].

##### Lipid synthesis

Lipids are significant components of vitreous warts, which are a specific manifestation of dry AMD [49]. Observational studies have considered HDL as a risk factor for AMD [50], however, the clinical importance of other lipids including LDL and triglycerides is still debatable owing to the limitation of sample sizes and confounding variables [51]. A direct causal association between HDL and AMD was found, with OR of 1.17 in Europeans and 1.58 in Asians, according to an analysis of genetic data from 41,270 participants (33,976 in Europe and 7,494 in Asia). However, there was no correlation between LDL, triglycerides and AMD [52]. According to a two-sample MR analysis, HDL was considered as a risk factor for AMD (OR 1.22) and its correlative gene regions cholesterol transfer protein (CETP) and apolipoprotein E (ApoE) would also increase AMD risk by increasing HDL concentrations [53].

##### Refractive error

Refractive error is associated with early and intermediate AMD with a low OR (OR < 1) but not advanced AMD, according to a research related to refractive error and AMD [54, 55]. Other researches have demonstrated that myopia is a protective factor against AMD while hyperopia is positively associated with its development, meaning that the potential risk of AMD is hyperopia > emmetropia > myopia [56–59]. Its prevalence might be influenced by scleral stiffness, endothelial cell growth factor concentration, and ocular axis length. A two-sample MR study showed a negligible causal relationship between AMD, myopia, and hyperopia and revealed that per diopter of hyperopia was associated with an OR of 1.080 in AMD [60]. These findings imply that individuals with refractive error, especially those with hyperopia, should be aware of their fundus health and have regular examinations.

##### Others

Additionally, a two-sample MR analysis conducted by Kuan et al. revealed that smoking and alcohol consumption raises the risk of AMD, whereas smoking cessation lowers it [61]. And a GWAS by Han et al. revealed that the prevalence of AMD in all subtypes rises with elevated serum C-reactive protein [62]. These findings shed light on additional susceptibility factors and make contributions to AMD prevention.

#### Diabetic retinopathy

One of the most prevalent microvascular complications of diabetes mellitus is diabetic retinopathy (DR), which can be classified as non-proliferative, proliferative and other risk factors such as hypertension, hyperglycemia, dyslipidemia, and others [63].The principal therapies are ranibizumab, whole retina laser photocoagulation, and anti-VEGF medications [64]. Ranibizumab-treated individuals had a decreased incidence of macular edema vision loss and visual field abnormalities, according to a research with a 5-year follow-up [65]. Currently, MR is mainly used in the study of DR pathogenesis. A bidirectional two-sample MR analysis revealed that two upstream regulators, IL-8 and SCGFb, and six downstream regulators including GROa, SDF1a, MCP3, GCSF, IL-12P70, and IL-2ra, increase the risk of developing PDR [66]. Skol et al. identified the FLCN locus as a susceptibility gene for DR and the glucose-responsive region as being related to DR via MR analysis [67]. Through an MR study, Liu et al. set out with intestinal microbes and concluded that the flora of Christiaceae and Digestive Coccidiaceae were causally connected with the pathogenesis of DR [68]. These underlying causes may develop into biomarkers for DR and offer more therapeutic design options.

### Ocular nerve disorders

#### Neuromyelitis Optica

Optic neuromyelitis optica (NMO) is a demyelinating disease that can be acute or subacute and affect the optic nerves and spinal cord simultaneously or in sequence. It is characterized by ocular symptoms like decreased vision and eye pain as well as spinal myelopathy symptoms like limb weakness, urinary and fecal disturbances. The term “optic neuromyelitis optica spectrum disorders” (NMOSDs) refers specifically to a group of related disorders with an underlying pathogenesis similar to NMO but with limited clinical involvement and only some of the defining manifestations. Since antibodies to aquaporin 4 (AQP4) are expected to attack one’s astrocytes in NMO, an autoimmune illness with an unclear etiology. Thus, AQP4-IgG has been recognized as a particular biomarker for NMO and NMOSDs [69].

Researchers utilize MR analyses to assess the effect of exposure using genetic variations for etiological analysis since NMO and NMOSDs are uncommon medical conditions with little clinical case data and it is immoral to conduct appropriate RCTs investigations due to ethical constraints. To confirm a genetic vulnerability to COVID-19 and NMOSD, Sun et al. performed a series of MR analyses as a first investigation. However, more findings from appropriate observational studies are still required to confirm a direct connection between the COVID-19 phenotype and the development of NMOSD [70]. The effects of 29 lifestyle and dietary factors were assessed in 132 AQP4-positive NMOSD patients and 784 controls from relevant GWAS using methods like IVW, MR-Egger etc., and it turned out that eating oily fish and raw vegetables reduced the risk of AQP4-positive NMOSD [71]. By using a two-sample MR analysis, Jasiak-Zatonska et al. investigated the relationship between other autoimmune diseases and NMOSD. He came to the conclusion that autoimmune thyroid disease (AITD), systemic lupus erythematosus (SLE), and desiccation syndrome (SS) were causally associated with an increased risk of NMOSD, while NMOSD did not contribute to AITD, SLE, or SS. Therefore, NMOSD cannot cause an increased risk of prevalence of AITD, SLE and SS [69]. These findings contribute to a better understanding of the etiology of NMOSD and provide potential therapeutic targets and options for patients.

### Myopia

Refractive errors, including myopia, hyperopia, and astigmatism. The main factor contributing to 43% of all cases of vision impairment is uncorrected refractive error, particularly myopia, which surges around the world [72]. The prevalence of myopia among young individuals in East and Southeast Asia is as high as 80–90%, with 10–20% of them being highly myopic [73].

#### Education and myopia

Education has long been acknowledged as a significant contributing factor to myopia. Morgan et al. noted that MR studies have shown a causal relationship between years of education and increased prevalence of myopia, while RCTs have shown that a certain amount of time spent outside is also a significant protective factor [74]. In addition, mass use of electronic devices and parents with myopia are risk factors for myopia in individuals. By using a bidirectional MR analysis, Mountjoy et al. investigated the causative link between education level and refractive error, and ultimately reached the conclusion that per additional year of education resulted in -0.27 diopter [75]. There was not that much evidence proves that myopia lengthens the time spent in school, and Plotnikov et al. came to the conclusion from MR analyses that the relationship between hyperopia and education is nonlinear and that mild hyperopia has no impact on the primary level of education. Instead, they found that myopia becomes more common as education is prolonged [76].

#### Others

Other factors have been hypothesized to be possibly related to the development of myopia, in addition to educational factors. Li et al. used a two-sample MR analysis and suggested that rigorous glycemic control can prevent the development of myopia. They discovered that decreasing levels of lipofuscin and rising levels of HbA1C increase the incidence of myopia [77]. Using SNPs associated with 25(OH)D levels as instrumental variables, Cuellar-Partida et al. investigated whether vitamin D insufficiency itself affects the development of myopia, disregarding the effect of outdoor activity from earlier research [78]. He found that vitamin D deficit was not linked to the degree of myopia eliminating interference from outdoor exercise. Additionally, a single-sample MR analysis revealed a minor but causal relationship between low birth weight and myopia promotion [79]. The summarizes of MR application in ocular diseases were shown in Table 1.

**Table 1:** MR analysis of ophthalmic related diseases mentioned in the references

Reference No.	Exposure	Correlation	MR method	OR/ *P*/95%CI
8.PMID: 36924037	Atopic dermatitis	Casual effect	Bidirectional two-sample MR	OR: Allergic conjunctivitis: 1.53 Atopic conjunctivitis: 1.76 Chronic conjunctivitis: 1.76
9.PMID: 37484661	ADHD	Not related	IVW etc.	OR: 0.99
11.PMID: 36159460	Omega-3 fatty acids Omega-6:3 fatty acids	Prevention Risk	Two-sample MR	OR: Omega-3 fatty acids 0.86 Omega-6:3 fatty acids 1.17
14.PMID: 35013517	Type2 diabetes mellitus High systolic blood pressure Smoking	Casual effects	MR	OR: BMI 1.19 SBP/10mmHg 1.13 T2D 1.06 Smoking 1.19
17.PMID: 19329528	Obesity	Significantly associated	MR	OR: Cortical 1.31 Posterior subcapsular 1.60
26.PMID: 33008364	LDL HDL TG	No evidence	IVW etc.	P: LDL 0.165 HDL 0.238 TG 0.206
28.PMID: 34838945	DHA AA etc.	No evidence	IVW etc.	OR: DHA 1.014 AA 0.993
31.PMID: 35537532	Caffeine intake	Positive	Two-sample MR	P: 0.0003
32.PMID: 33333105	Habitual caffeine consumption	Weakly associated	MR	OR: 1.155
35.PMID: 35900730	Myopia	Genetically correlated	Two-sample MR	P: Myopia 0.04 High myopia 0.01
36.PMID: 36493903	Myopia	Causal relationship	MR	P: 1.37 × 10^− 8^
38.PMID: 36835817	UC CD	Positively associated	Bidirectional MR	OR: CD 8.24 UC 3.29
39.PMID: 35974138	LA AA	Prevention Risk	MR	OR: LA 0.94 AA 1.053
40.PMID: 37396905	TIM-3	Protective factor	IVW	OR: 0.889
43.PMID: 33599689	Tuberculosis infection	Risk	IVW	OR: 2.26
44.PMID: 32593521	High 25(OH)D level	Risk	IVW	OR: 3.96
52.PMID: 29025108	HDL	Casual association	MR	OR: 1.53 (Asia) 1.17 (Europe)
53.PMID: 28456421	HDL	Risk factor	Two-sample MR	OR: 1.22
60.PMID: 30905725	Myopia Hyperopia	Negligible causal relationship	Two-sample MR	OR: 1.08
61.PMID: 34734970	Smoking Alcohol consumption	Risk	Two-sample MR	OR: Smoking 1.32 Alcohol consumption 2.70
62.PMID: 31900758	C-reactive protein	Risk	Two-sample MR	OR: 1.31
66.PMID: 36845092	SCGFb IL-8	Risk	Bidirectional two-sample MR	95%CI: SCGFb 3.8% 41.9% IL-8 0.6% 24.2%
68.PMID: 36159877	Christensenellaceae Peptococcaceae	Risk	IVW etc.	P: Christensenellaceae 1.36 × 10^− 2^ Peptococcaceae 3.13 × 10 − 2
70.PMID: 37575255	COVID-19	Risk	MR	OR: 4.958
71.PMID: 37296187	Oily fish Raw vegetables	Prevention	IVW etc.	OR: 1.78 × 10^− 16^
75.PMID: 29875094	Education level	Risk	Bidirectional MR analysis	95%CI: -0.18D -19% -17% -0.27D -37% -17%
76.PMID: 34709397	Hyperopia	Nonlinearity	MR	P: 8.8e-0.5
59.PMID: 36867130	Lipofuscin HbA1C	Protection Risk	IVW etc.	OR: Lipofuscin 0.99 HbA1C 1.02
78.PMID: 28586461	25(OH)D	Not linked	MR	95%CI: -9% 4%
79.PMID: 31097437	Low birth weight	Positive association	Single-sample MR	95%CI: 5% 52%

## Summary and outlook

MR for guiding etiological studies is booming and opening significant new opportunities to identify environmental exposures that increase or decrease the risk of ocular diseases, but it still has its limitations to be improved.

 (1) Linkage disequilibrium (LD): LD is a condition that causes alleles to segregate in a way that deviates from Mendel’s second law, which states that not all non-alleles will mix easily. This leads to disturbances between related genetic variations, i.e. the likelihood of an eventual bias is contingent upon the association between the outcome and the IVs. This issue can be resolved by using MR in populations with various LD structures.

 (2) Gene pleiotropy is the situation in which a single locus influences two or more phenotypic features. Of the two pleiotropy processes [80], type I pleiotropy, in which a single locus directly influences numerous phenotypes, may impede the results of research due to the intimate connection [2]. And it can be detected and corrected by sensitivity analysis.

 (3) Differences in ethnicities: For MR analysis, genetic information is frequently taken from biobanks with a focus on a particular region (such as the UK Biobank). The conclusions reached may range substantially depending on the subject populations’ allele frequencies and the prevalence of linked disorders. Large inaccuracies may exist when IV, exposure, and outcome are collected from different groups. In practice, this can be avoided by restricting the background of the subject population. If this is not possible, adaptive switching can be performed first to minimize the error.

Despite our understanding of the risk factors for ocular diseases has advanced significantly, effective prevention and treatments have not yet been effectively implemented. The pathophysiology of complicated disorders and associated translational research such as the drug target identification and gene therapy, which plays a significant role in clinical practice are absent from the current MR research in ophthalmology. In light of these, finding drug targeted proteins and utilizing genes as instrumental variables by combining GWAS with multi-omics data, such as proteomics and metabolomics, may prove to be a novel approach for the application of MR in ophthalmology.

## Data Availability

No datasets were generated or analysed during the current study.

## References

[1] Sekula P, Del Greco M, Pattaro F, Köttgen A (2016). Mendelian randomization as an Approach to assess causality using Observational Data. J Am Soc Nephrol JASN.

[2] Emdin CA, Khera AV, Kathiresan S (2017). Mendelian Randomization JAMA.

[3] Davey Smith G, Ebrahim S (2005). What can mendelian randomisation tell us about modifiable behavioural and environmental exposures?. BMJ.

[4] Kang YT, Li SM (2021). [Application of mendelian randomization in ophthalmology and other medical fields]. Zhonghua Yan Ke Za Zhi Chin J Ophthalmol.

[5] Bowden J, Davey Smith G, Burgess S (2015). Mendelian randomization with invalid instruments: effect estimation and bias detection through Egger regression. Int J Epidemiol.

[6] Miyazaki D (2020). Epidemiological aspects of allergic conjunctivitis. Allergol Int off J Jpn Soc Allergol.

[7] von Mutius E (2000). International patterns of tuberculosis and the prevalence of symptoms of asthma, rhinitis, and eczema. Thorax.

[8] Zhou W, Cai J, Li Z, Lin Y (2023). Association of atopic dermatitis with conjunctivitis and other ocular surface diseases: a bidirectional two-sample mendelian randomization study. J Eur Acad Dermatol Venereol JEADV.

[9] Zhang X, Zhang R, Zhang Y, Lu T (2023). Associations between attention-deficit/hyperactivity disorder and allergic diseases: a two-sample mendelian randomization study. Front Psychiatry.

[10] Liu K, Cai Y, Song K, Yuan R, Zou J (2023). Clarifying the effect of gut microbiota on allergic conjunctivitis risk is instrumental for predictive, preventive, and personalized medicine: a mendelian randomization analysis. EPMA J.

[11] Li Y, Li Q, Cao Z, Wu J (2022). The causal association of polyunsaturated fatty acids with allergic disease: a two-sample mendelian randomization study. Front Nutr.

[12] Pascolini D, Mariotti SP (2012). Global estimates of visual impairment: 2010. Br J Ophthalmol.

[13] Purola PKM (2022). Prevalence and 11-Year incidence of cataract and cataract surgery and the effects of Socio-demographic and lifestyle factors. Clin Ophthalmol Auckl NZ.

[14] Yuan S, Wolk A, Larsson SC (2022). Metabolic and lifestyle factors in relation to senile cataract: a mendelian randomization study. Sci Rep.

[15] Jiang C (2023). Association of behavioral and clinical risk factors with cataract: a two-sample mendelian randomization study. Invest Ophthalmol Vis Sci.

[16] Hiller R, Sperduto RD, Ederer F (1986). Epidemiologic associations with nuclear, cortical, and posterior subcapsular cataracts. Am J Epidemiol.

[17] Lim LS (2009). Relation of age-related cataract with obesity and obesity genes in an Asian population. Am J Epidemiol.

[18] Quigley HA, Broman AT (2006). The number of people with glaucoma worldwide in 2010 and 2020. Br J Ophthalmol.

[19] Bluwol E, [Glaucoma, treatment]. Rev Prat. 2016;66:508–13. 30512573

[20] Weinreb RN, Aung T, Medeiros FA (2014). The pathophysiology and treatment of glaucoma: a review. JAMA.

[21] Kang JM, Lin S (2018). Ginkgo biloba and its potential role in glaucoma. Curr Opin Ophthalmol.

[22] McMonnies C (2018). Reactive oxygen species, oxidative stress, glaucoma and hyperbaric oxygen therapy. J Optom.

[23] Yokomichi H (2016). Evaluation of the associations between changes in intraocular pressure and metabolic syndrome parameters: a retrospective cohort study in Japan. BMJ Open.

[24] Perez CI, Singh K, Lin S (2019). Relationship of lifestyle, exercise, and nutrition with glaucoma. Curr Opin Ophthalmol.

[25] Newman-Casey PA, Talwar N, Nan B, Musch DC, Stein JD (2011). The relationship between components of metabolic syndrome and open-angle glaucoma. Ophthalmology.

[26] Xu M (2020). Plasma lipid levels and risk of primary open angle glaucoma: a genetic study using mendelian randomization. BMC Ophthalmol.

[27] Nusinovici S (2022). High-density lipoprotein 3 cholesterol and primary Open-Angle Glaucoma: Metabolomics and mendelian randomization analyses. Ophthalmology.

[28] Bao J, Yang Z, Zheng S, Li J, Shentu X (2022). Circulating fatty acids and risk of primary open-angle glaucoma: a mendelian randomization study. Gene.

[29] Vera J, Redondo B, Molina R, Bermúdez J, Jiménez R (2019). Effects of caffeine on intraocular pressure are subject to tolerance: a comparative study between low and high caffeine consumers. Psychopharmacology.

[30] Jiwani AZ (2012). Effects of caffeinated coffee consumption on intraocular pressure, ocular perfusion pressure, and ocular pulse amplitude: a randomized controlled trial. Eye Lond Engl.

[31] Li X (2022). Habitual Coffee Consumption increases risk of primary Open-Angle Glaucoma: a mendelian randomization study. Ophthalmology.

[32] Kim J (2021). Intraocular pressure, Glaucoma, and Dietary Caffeine Consumption: a Gene-Diet Interaction Study from the UK Biobank. Ophthalmology.

[33] Marcus MW, de Vries MM, Montolio J, F. G., Jansonius NM. Myopia as a risk factor for open-angle glaucoma: a systematic review and meta-analysis. Ophthalmology 118, 1989–1994.e2 (2011). 10.1016/j.ophtha.2011.03.012 21684603

[34] Ha A, Kim CY, Shim SR, Chang IB, Kim YK (2022). Degree of myopia and Glaucoma risk: a dose-response Meta-analysis. Am J Ophthalmol.

[35] Choquet H (2022). Association between myopic refractive error and primary Open-Angle Glaucoma: a 2-Sample mendelian randomization study. JAMA Ophthalmol.

[36] Chong RS (2023). Mendelian Randomization Implicates Bidirectional Association between Myopia and Primary Open-Angle Glaucoma or intraocular pressure. Ophthalmology.

[37] Huang X-F, Brown MA (2022). Progress in the genetics of uveitis. Genes Immun.

[38] Meng Y, Tan Z, Liu C, Dong W, Chen C (2023). Association between Inflammatory Bowel Disease and Iridocyclitis: a mendelian randomization study. J Clin Med.

[39] Shu Q (2023). Causal analysis of serum polyunsaturated fatty acids with juvenile idiopathic arthritis and ocular comorbidity. Eur J Clin Nutr.

[40] Lin D (2023). Association of TIM-3 with anterior uveitis and associated systemic immune diseases: a mendelian randomization analysis. Front Med.

[41] Gul A, Ohno S (2012). HLA-B*51 and Behçet Disease. Ocul Immunol Inflamm.

[42] Bulur I, Onder M (2017). Behçet disease: new aspects. Clin Dermatol.

[43] Zhong Z (2021). Tuberculosis exposure with risk of Behçet Disease among patients with Uveitis. JAMA Ophthalmol.

[44] Zhong Z (2021). Higher 25-hydroxyvitamin D level is associated with increased risk for Behçet’s disease. Clin Nutr Edinb Scotl.

[45] Tong B, Liu X, Xiao J, Su G (2019). Immunopathogenesis of Behcet’s Disease. Front Immunol.

[46] van Lookeren Campagne M, LeCouter J, Yaspan BL, Ye W (2014). Mechanisms of age-related macular degeneration and therapeutic opportunities. J Pathol.

[47] Ricci F (2020). Neovascular age-related Macular Degeneration: Therapeutic Management and New-Upcoming approaches. Int J Mol Sci.

[48] de Cabral TA, Daich Varela M, Georgiou M, Michaelides M (2022). Treatments for dry age-related macular degeneration: therapeutic avenues, clinical trials and future directions. Br J Ophthalmol.

[49] Lin JB, Halawa OA, Husain D, Miller JW, Vavvas DG (2022). Dyslipidemia in age-related macular degeneration. Eye Lond Engl.

[50] Wang Y (2016). The Association between the lipids levels in blood and risk of age-related Macular Degeneration. Nutrients.

[51] Sim RZH (2022). Relationships between lipid-related metabolites and age-related Macular Degeneration vary with complement genotype. Ophthalmol Sci.

[52] Fan Q (2017). HDL-cholesterol levels and risk of age-related macular degeneration: a multiethnic genetic study using mendelian randomization. Int J Epidemiol.

[53] Burgess S, Davey Smith G (2017). Mendelian randomization implicates high-density lipoprotein cholesterol-Associated mechanisms in etiology of age-related Macular Degeneration. Ophthalmology.

[54] Lee K, Kwon J-W, Jahng WJ, Park Y-H, Jee D (2020). Age- and sex-based evaluation of the association between refractive error and age-related macular degeneration in the Korean population. PLoS ONE.

[55] Lin S-C, Singh K, Chao DL, Lin SC (2016). Refractive error and the risk of age-related Macular Degeneration in the South Korean Population. Asia-Pac J Ophthalmol Phila Pa.

[56] Pan C-W (2013). Refractive errors and age-related macular degeneration: a systematic review and meta-analysis. Ophthalmology.

[57] Lavanya R (2010). Hyperopic refractive error and shorter axial length are associated with age-related macular degeneration: the Singapore malay Eye Study. Invest Ophthalmol Vis Sci.

[58] Quigley MG, Powell I, Wittich W (2018). Increased axial length corresponds to decreased retinal light dose: a parsimonious explanation for decreasing AMD Risk in Myopia. Invest Ophthalmol Vis Sci.

[59] Pan C-W (2013). Differential associations of myopia with major age-related eye diseases: the Singapore Indian Eye Study. Ophthalmology.

[60] Wood A, Guggenheim JA (2019). Refractive error has minimal influence on the risk of age-related Macular Degeneration: a mendelian randomization study. Am J Ophthalmol.

[61] Kuan V (2021). Association of Smoking, Alcohol Consumption, blood pressure, body Mass Index, and glycemic risk factors with age-related Macular Degeneration: a mendelian randomization study. JAMA Ophthalmol.

[62] Han X (2020). Using mendelian randomization to evaluate the causal relationship between serum C-reactive protein levels and age-related macular degeneration. Eur J Epidemiol.

[63] Cheung N, Mitchell P, Wong TY (2010). Diabetic retinopathy. Lancet Lond Engl.

[64] Lin K-Y, Hsih W-H, Lin Y-B, Wen C-Y, Chang T-J (2021). Update in the epidemiology, risk factors, screening, and treatment of diabetic retinopathy. J Diabetes Investig.

[65] Gross JG (2018). Five-year outcomes of Panretinal Photocoagulation vs Intravitreous Ranibizumab for proliferative Diabetic Retinopathy: a Randomized Clinical Trial. JAMA Ophthalmol.

[66] Shi Q, Wang Q, Wang Z, Lu J, Wang R (2023). Systemic inflammatory regulators and proliferative diabetic retinopathy: a bidirectional mendelian randomization study. Front Immunol.

[67] Skol AD (2020). Integration of genomics and transcriptomics predicts diabetic retinopathy susceptibility genes. eLife.

[68] Liu K, Zou J, Fan H, Hu H, You Z (2022). Causal effects of gut microbiota on diabetic retinopathy: a mendelian randomization study. Front Immunol.

[69] Jasiak-Zatonska M, Kalinowska-Lyszczarz A, Michalak S, Kozubski W (2016). The immunology of Neuromyelitis Optica-current knowledge, clinical implications, controversies and future perspectives. Int J Mol Sci.

[70] Sun D (2023). COVID-19 and the risk of neuromyelitis optica spectrum disorder: a mendelian randomization study. Front Immunol.

[71] Wang S (2023). Oily fish and raw vegetable consumption can decrease the risk of AQP4-positive neuromyelitis optica spectrum disorders: a mendelian-randomization study. Sci Rep.

[72] Pascolini D, Mariotti SP (2012). Global estimates of visual impairment: 2010. Br J Ophthalmol.

[73] Morgan IG (2018). The epidemics of myopia: Aetiology and prevention. Prog Retin Eye Res.

[74] Morgan IG (2021). IMI Risk factors for myopia. Invest Ophthalmol Vis Sci.

[75] Mountjoy E et al. Education and myopia: assessing the direction of causality by mendelian randomisation. *BMJ* 361, k2022 (2018). 10.1136/bmj.k2022 PMC5987847 29875094

[76] Plotnikov D (2021). Hyperopia is not causally Associated with a major deficit in Educational Attainment. Transl Vis Sci Technol.

[77] Li F-F (2023). Causal relationships between glycemic traits and myopia. Invest Ophthalmol Vis Sci.

[78] Cuellar-Partida G (2017). Genetically low vitamin D concentrations and myopic refractive error: a mendelian randomization study. Int J Epidemiol.

[79] Plotnikov D, Williams C, Guggenheim JA (2020). Association between birth weight and refractive error in adulthood: a mendelian randomisation study. Br J Ophthalmol.

[80] Wagner GP, Zhang J (2011). The pleiotropic structure of the genotype-phenotype map: the evolvability of complex organisms. Nat Rev Genet.

